# Malocclusions and Dental Diseases in Privately Owned Horses in the Mazovia Region of Poland

**DOI:** 10.3390/ani12223120

**Published:** 2022-11-11

**Authors:** Kamil Górski, Elżbieta Stefanik, Bernard Turek, Andrzej Bereznowski, Michał Czopowicz, Izabela Polkowska, Małgorzata Domino

**Affiliations:** 1 Department of Large Animal Diseases and Clinic, Institute of Veterinary Medicine, Warsaw University of Life Sciences, 02-787 Warsaw, Poland; 2 Division of Veterinary Epidemiology and Economics, Institute of Veterinary Medicine, Warsaw University of Life Sciences, Nowoursynowska 159c, 02-776 Warsaw, Poland; 3 Department and Clinic of Animal Surgery, Faculty of Veterinary Medicine, University of Life Sciences in Lublin, 20-950 Lublin, Poland

**Keywords:** equine dentistry, dental disorders, malocclusion, occurrence, horse

## Abstract

**Simple Summary:**

Dental care is an integral part of equine veterinary practice and has a significant impact on the welfare and body condition score of horses. Regular and detailed examination of the oral cavity allows for diagnosis of malocclusions and dental disease and the implementation of appropriate treatment. As predispositions for individual dental diseases differ among horse populations and age groups, this study aims to characterize the prevalence and frequency distribution of selected malocclusions and dental diseases among horses housed in the Mazovia region of Poland, using the prevalence and frequency distribution of selected malocclusions and dental diseases. Routine veterinary dental examinations were carried out on 206 horses, and the presenting signs of specific malocclusions and dental diseases were recorded for the incisor, canine, wolf, premolar, and molar teeth. Ninety-five percent of examined horses presented with a dental disorder in at least one tooth, with malocclusions more prevalent than dental disease in the incisors, premolars, and molars alike. Curvatures and calculus were the most commonly reported pathologies in incisor teeth, whereas sharp enamel points and caries predominated in cheek teeth.

**Abstract:**

Dental disorders, a term encompassing both malocclusion and dental disease, constitute a serious health problem in horses worldwide. As horse populations differ among countries and regions, a geographically specific characterization of the occurrence of various dental disorders may be helpful for local equine practitioners. This study investigated the prevalence and frequency distribution of selected malocclusions and dental diseases in horses housed in the Mazovia region of Poland, with attention paid to variations among age, gender, and breed categories. Routine dental examinations were conducted on 206 privately owned horses (*n* = 206). Disorders were recorded using a dental chart and classified as either malocclusions or dental diseases. Out of all examined horses, 95% demonstrated at least one dental disorder, with a roughly equal distribution of these disorders among incisor teeth (31%) and cheek teeth (31% each for premolars and molars). More specifically, there were disorders noted in 14 incisors, 3 canines, 2 wolf teeth, and 15 cheek teeth. Across all age, gender, and breed groups, malocclusions of incisor, premolar, and molar teeth occurred with a higher prevalence than did dental diseases. Curvatures and calculus were the most commonly reported pathologies in incisor teeth, whereas sharp enamel points and caries predominated in cheek teeth.

## 1. Introduction

It is widely accepted that oral cavity diseases and their sequalae constitute a serious health problem in horses worldwide [1]. Both routine [2,3,4] and post mortem [5,6] examinations of the oral cavity have revealed that dental diseases are the most commonly diagnosed oral cavity pathology. As previously indicated by Salem et al. [7], most studies of equine dental disease type and incidence are based on clinical cases of hospitalized horses or autopsies of equine heads obtained from slaughterhouses [3,5,8,9,10]. Kirkland [11] examined 500 horse skulls isolated from cadavers and confirmed 80% showed evidence of dental problems. In a similar but smaller post mortem study of 50 skulls, Brigham et al. found a 74% rate of dental disease [3]. In North America, dental diseases are among the five most common health problems in adult horses [12], affecting 79% of the local horse population [6]. In England, 42% of owners reported their horse suffered from a known dental disorder [13]. In Scotland, 87% of the local horse population is suspected to have a dental disease [14]. In Australia, out of 400 horses referred to clinics due to dental complaints, 87% had a primary disorder in the area of the cheek teeth, 13% had a serious disorder resulting from significant tooth wear, and 11% did not show clinical signs [15]. Out of 400 horse heads examined in an Australian slaughterhouse, 94% showed at least one tooth defect [15]. Similarly, when 556 horse cadavers were examined in a Canadian slaughterhouse, 70% had at least one type of dental abnormality in the cheek teeth [12].

In Poland, apart from one study documenting the frequency of incisor defects [16], the incidence of equine dental abnormalities has not yet been reported. However, given the prevalence of dental diseases in other countries and the horse population in Poland, one can expect to find similar rates of occurrence in Polish horses. According to data provided by the Polish Horse Breeders Association (PHBA-PZHK), there were 271,324 horses in Poland in 2019, including 131,661 cold-blooded horses, 93,852 Polish sport horses, and 45,881 ponies [17]. Among these horses, 37,397 were registered in the Mazovian Voivodeship [18] as sport horses associated with equestrian centers [19] or were privately owned and housed on small horse farms [18]. According to the same dataset, 111 equestrian centers were registered in Mazovia Voivodeship, with each housing an average of 50 horses [19]. It can therefore be estimated that about 5550 horses in Mazovia are sport horses under specific veterinary care, while the remaining 31,847 horses are privately owned. According to the data provided by the Central Statistical Office (CSO-GUS), there are 4550 small horse farms registered in Mazovia, with an average of 7 horses stabled at each. Among the 5,358,000 people of Mazovia Voivodeship, it is estimated 0.08% keep horses for pleasure [18]. As the majority of horses in the region (~85%) are privately owned, the privately owned horses included in this study constitute a reasonably representative group. Moreover, in accordance with veterinary guidelines [7], all privately owned horses should undergo a routine dental examination once a year to determine the health status of the teeth and to perform any necessary corrections. Such annual examinations are advised due to the continuous growth rate of equine teeth and the anisognathic mandible and maxilla conformation, which can lead to irregular wear [1,6].

Examination of dental charts of horses presented for routine dentistry over the years reveal an increase in the percentage of horses manifesting dental problems. Disorders of the incisor teeth, for example, were diagnosed in 11% of horses in the study in 1999 [20], in 20% in 2005 [21], and in 26% in 2008 [22]. One of the reasons for the observed increased incidence of dental disease is improved recognition, due to greater acceptance among owners of the importance of a regularly scheduled dental examination [23], the availability of advanced diagnostic imaging, such as computed tomography (CT) and magnetic resonance imaging (MRI) [24,25], as well as the increased proclivity of veterinarians to specialize in equine dentistry [25,26,27]. In light of the growing understanding of the impact of proper dental care on the horse’s ability to properly chew and grind fodder [3], on equine welfare [28,29], and on sport and working horse usage [30,31,32], emphasis on odontology as a means of diagnosing, treating, and preventing oral cavity diseases is also increasing [27].

Dental diseases are generally painful [33,34], and thus greatly impact both the horses’ quality of life [28] and the owner’s ability to use the animal [31,32]. Consequences of dental disease such as pain, discomfort, and altered feed intake have a negative impact on a horse’s well-being and are part of the equine welfare monitoring system assessment protocol [29]. This protocol requires the assessor to note the presence of any abnormal wear of the incisors, which might negatively affect feed intake, and asks with what frequency the horse’s teeth are being inspected by an equine dental veterinarian [29]. With respect to horse usage, wolf teeth and hooks of the premolar teeth may interfere with the bit, affecting rein tension-related response and consequently the ride ability of the horse [31]. If wolf teeth are present and cause biting issues, they should be removed as early as possible in the horse’s career in order to reduce training disturbances [31]. Similarly, if hooks are present, especially on the upper second premolars, they should be corrected to avoid discomfort associated with the bit [31]. In domestic sport and working horses, erosive lesions and fractures of the lower second premolar dental hard tissues and canine teeth are common [30,32]. When dental disease is diagnosed, specific treatment and often a break in riding, training, and competition is required. Moreover, in the case of aggravations or complications, treatment is prolonged, and the financial costs are significantly higher [35]. Thus, both the outright financial losses for the owner and the disruption to the horse’s training plan make dental disease an important issue in the equine industry.

Salem et al. [7] highlighted the integral role of field investigations in determining the prevalence of oral cavity and dental disorders and noted that these data contribute to veterinarian and owner knowledge regarding the impact of dental disease on horse health and welfare. Therefore, this study describes the prevalence and frequency distribution of selected malocclusions and dental diseases in a population group of privately owned horses housed in the Mazovia region of Poland, taking into consideration age, gender, and breed.

## 2. Materials and Methods

### 2.1. Horses

The study population includes 206 privately owned horses (*n* = 206) (age mean ± SD: 16.9 ± 7.0; 114 geldings, 78 mares, 14 stallions), presented to a dental veterinarian by their owners for the annual routine dental examination between January and December 2019. The horses were housed in a total of 45 different private stables, all located in Mazovia Voivodeship in Poland, with each stable contributing an average of 4.6 horses to the study. Therefore, horses from 1% (*n* = 45) of private stables located in the studied region (*n* = 4550) were examined. At each stable, all horses were inspected as part of a routine annual dental check-up, independent of oral health status. Owners provided written consent to the inclusion of their horses’ data in the current study.

The prevalence of malocclusions and dental disease was considered with respect to three variables: age, gender, and breed. The horses were divided into four age groups: (i) 0–5 years old (*n* = 20; 9 geldings, 6 stallions, 5 mares), (ii) 6–10 years old (*n* = 75; 45 geldings, 4 stallions, 26 mares), (iii) 11–15 years old (*n* = 57; 30 geldings, 3 stallions, 24 mares), and (iv) > 15 years old (*n* = 54; 30 geldings, 1 stallion, 23 mares). Additionally, horses were assigned to one of two gender groups: males (*n* = 128; 114 geldings, 14 stallions) and females (*n* = 78; 78 mares). Finally, the horses, all from warm-blooded breeds, were divided into three sub-groups: (i) Polish warmblood (*n* = 109; 69 geldings, 4 stallions, 36 mares), (ii) pony (*n* = 40; 22 geldings, 3 stallions, 15 mares), and (iii) “other” (*n* = 57; 23 geldings, 7 stallions, 27 mares). The Polish warmblood group comprised four breeds: the Polish Halfbred (*n* = 73), the Wielkopolska (*n* = 10), the Malopolska (*n* = 17), and Schlesisches Warmblood (*n* = 9). The pony group included warm-blooded mixed-breed ponies (*n* = 27), Arabian horses (*n* = 5), Carpathian ponies (*n* = 4), Welsh ponies (*n* = 1), Welsh ponies (*n* = 2), and Halfinger ponies (*n* = 2). The “other” group included warm-blooded mix-breeds (*n* = 24), Thoroughbreds (*n* = 15), Spanish Warmbloods (*n* = 7), Dutch Warmbloods (*n* = 4), Hanoverians (*n* = 4), and Holsteiners (*n* = 3).

As the dental examinations were standard veterinary diagnostic procedures, no ethical approval was required.

### 2.2. Dental Examination

The veterinary diagnostic procedure began with a basic clinical examination. The internal temperature, heart rate, respiratory rate, mucous membrane color and moistness, capillary refill time, and lymph nodes were evaluated in accordance with international veterinary standards [36]. A detailed examination of the oral cavity was performed following the relevant professional guidelines [7,37,38].

In order to facilitate a complete oral, dental, and radiological examination, horses were sedated with detomidine hydrochloride (Domosedan; Orion Corporation, Espoo, Finland; 0.01 mg/kg bwt i.v.), xylazine hydrochloride (Xylapan; Vetoquinol Biowet Sp. Zo.o., Gorzów Wielkopolski, Poland; 0.4 mg/kg bwt i.v.), or a combination of both, with some requiring the additional of butorphanol tetrate (Torbugesic; Zoetis Polska Sp. z o.o., Warsaw, Poland; 0.01 mg/kg bwt i.v.). The dose and composition of the sedation was determined on the basis of the horse’s body weight and temperament.

**Figure 1:**
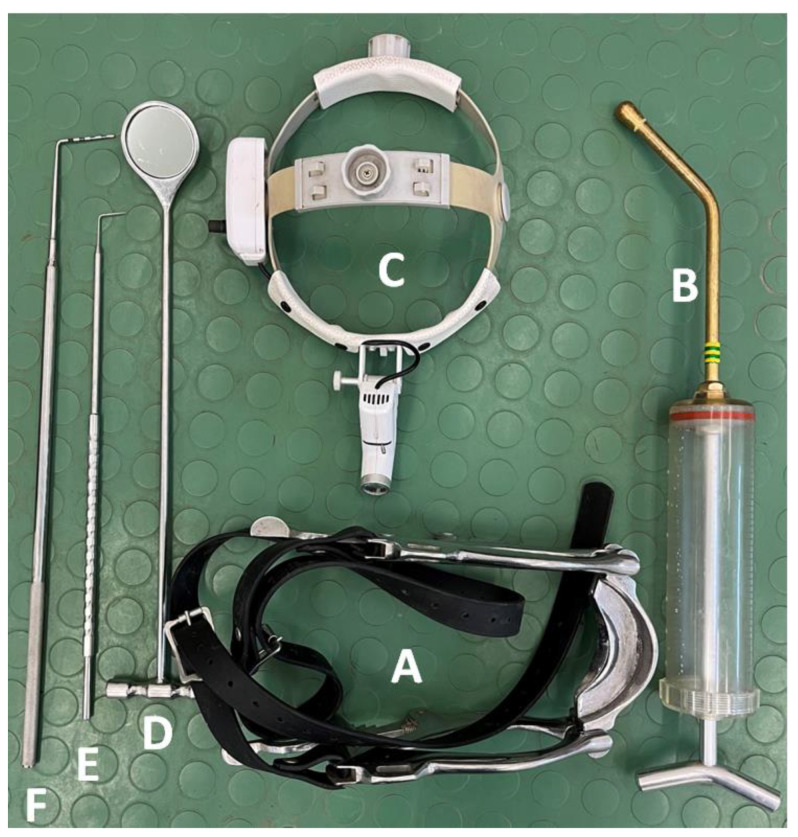
The equipment used in a detailed dental examination. Haussmann’s mouth speculum (**A**); 400 mL syringe (**B**); headlamp (**C**); dental mirror (**D**); dental hook (**E**); and periodontal probe (**F**).

A detailed dental examination was performed using the following equipment: Haussmann’s mouth speculum (to open the oral cavity for visual examination and digital palpation); a 400 mL syringe (to rinse the oral cavity with water and remove the remaining food); a headlamp (for illumination of the oral cavity during visual examination); a dental mirror (for more accurate observation of occlusal changes and for examination of the interdental spaces, the condition of the gums, and the mucosa of the cheeks and tongue, especially in the caudal part of the oral cavity); a periodontal probe (to examine the interdental spaces); and a dental hook (to examine the occlusal surfaces of the teeth) (Figure 1).

The detailed dental examination included both visual observation and digital palpation, and the findings were recorded using a dental chart presented in Supplementary Figure S1 and available online. The equine dental nomenclature was unified following the modified Triadan system [39]. In the maxilla, the right-hand quadrant was identified as “1” and the left-hand quadrant as “2”. Teeth were numbered consecutively beginning with 01 at the midline and proceeding distally. In the mandible, the left-hand quadrant was identified as “3” and the right-hand as “4”, and the teeth were numbered as in the maxilla.

Oral cavity inspection began with a visual examination and digital palpation of the incisor teeth (upper right: 101, 102, 103; upper left: 201, 202, 203; lower left: 301, 302, 303; lower right: 401, 402, 403) [39]. The line created by the occlusal surfaces of the maxillary and mandibular incisors was evaluated. The occurrence of underbite or overbite, as well as ventral, dorsal, diagonal, or irregular curvature was noted. Missing or supernumerary teeth, retained milk teeth, and diastemata were marked. Additionally, indications of Equine Odontoclastic Tooth Resorption and Hypercementosis (EOTRH) syndrome, such as periodontal inflammation, gingivitis of different degrees and gingival recession, denuding of lamina dura dentis, deposition of tooth calculus, accumulation of food remains, tooth instability, dental abscesses, and periodontal fistulation were evaluated [34,37].

Next, visual examination and digital palpation of any canine teeth (upper right: 104; upper left: 204; lower left: 304; lower right: 404) [39] was performed. Calculus deposition and associated gingivitis, canine fracture, peripheral caries and non-erupted canines were considered [38].

Subsequently, the Hausmann speculum was utilized to open the oral cavity, which was then rinsed with plenty of water using a drencher. After rinsing, visual examination and digital palpation of the cheek teeth was performed, including: the vestigial first premolars (wolf teeth; upper right: 105; upper left: 205; lower left: 305; lower right: 405); premolar teeth (upper right: 106, 107, 108; upper left: 206, 207, 208; lower left: 306, 307, 308; lower right: 406, 407, 408), and molar teeth (upper right: 109, 110, 111; upper left: 209, 210, 211; lower left: 309, 310, 311; lower right: 409, 410, 411) [39]. A detailed assessment of the occlusal surface of the cheek teeth was facilitated by use of a dental mirror and the direct light of a headlamp. All tooth surfaces—buccal, palatine, lingual, labial, and occlusal [15]—were assessed. Diastema, retained milk teeth fragments, fractures, infandibulum and peripheral caries, calculus, gingivitis, gum recession and periodontal pockets, erosions, ulcers, as well as lacerations of the buccal or lingual mucous membrane were evaluated [40,41]. Moreover, the tongue was examined for signs of any possible inflammation or trauma, and oral mucous membranes were examined for signs of injuries such as ulcerations, buccal calluses (loss of superficial epithelium with evidence of necrosis or areas of mucosal epithelial hyperplasia) and/or lacerations (tears in the oral mucosa). Lingual scarring was defined as evidence of old lacerations or other healed tongue injuries with resultant scar tissue formation, e.g., due to inappropriate bits or use of tongue twitches [7].

### 2.3. Malocclusions and Dental Disease Classification

**Figure 2:**
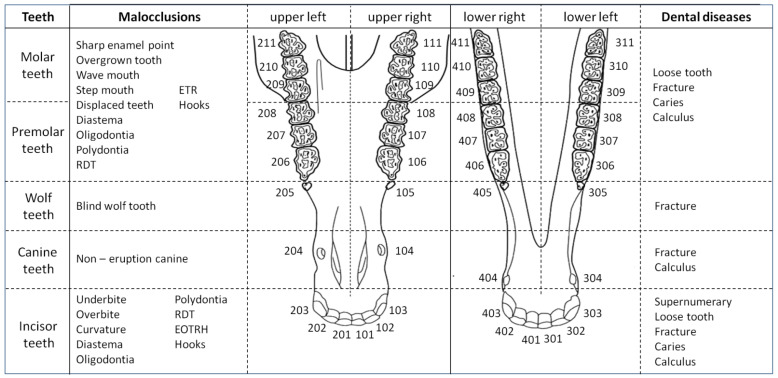
Classification of the malocclusions and dental diseases of the incisor (101, 102, 103, 201, 202, 203, 301, 302, 303, 401, 402, and 403); canine (104, 204, 304, and 404); wolf (105, 205, 305, and 405); premolar (106, 107, 108, 206, 207, 208, 306, 307, 308, 406, 407, and 408); and molar (109, 110, 111, 209, 210, 211, 309, 310, 311, 409, 410, and 411) teeth in horses. ETR—excessive transverse ridges; RDT—retained deciduous teeth; EOTRH—Equine Odontoclastic Tooth Resorption and Hypercementosis syndrome.

Based on the signs detected during the detailed dental examination, the following malocclusions (i) and dental diseases (ii) were diagnosed for the incisor, canine, wolf, premolar, and molar teeth, respectively (Figure 2). The definitions of selected disorders are summarized in Supplementary Tables S1–S4 available online.

For the incisor teeth, detected malocclusions (i) included the following: underbite, overbite, curvature (ventral, dorsal, slant, or irregular), diastema, oligodontia, polydontia, retained deciduous teeth (RDT), EOTRH, and hooks. In addition, dental diseases (ii) included the following: supernumerary teeth, loose teeth, fractures (marginal, transverse, sagittal, and undefined), caries (infundibular and peripheral), and calculus.

For the canine teeth, malocclusions (i) included non-erupted canines, and dental diseases (ii) included fractures (marginal, transverse, sagittal, and undefined) and calculus.

For the wolf teeth, malocclusions (i) included blind wolf teeth and dental disease (ii) included fractures.

The same malocclusions (i) and dental diseases (ii) are applicable to the premolar and molar teeth, and include the following: (i) sharp enamel points, overgrown teeth, wave mouth, step mouth, displaced teeth, diastema, oligodontia, polydontia, RDT, excessive transverse ridges (ETR), and hooks; (ii) loose teeth, fractures (marginal, transverse, sagittal, and undefined), caries (infundibular and peripheral), and calculus.

### 2.4. Statistical Analysis

The statistical analysis was conducted following the model used by Huang and Chen [42] to report the prevalence of signs of selected dental diseases in a limited population of people.

The prevalences of dental malocclusions and diseases were calculated for the population of studied horses as a whole, as well as for each sub-grouping according to age, gender, and breed. The data were evaluated by Chi-square analysis, where expected and observed values were entered as percentages representing malocclusions and dental disease distributions, respectively. The part of whole comparison was conducted twice, in relation to the whole horses’ group and concerning the number of affected teeth. Differences were considered significant when *p* < 0.05.

The frequency distributions of teeth malocclusions and dental diseases were calculated for the studied horse population in its entirety as well as for each individual sub-grouping according to the variables of age, gender, and breed. For the calculation of frequency distribution, each horse was represented as one realization where values <0; 12> for incisor, premolar, and molar teeth, <0; 4> for canine teeth, and <0; 2> for wolf teeth were assigned to each malocclusion or dental disease for individual teeth. Thus, each realization ranged from 0 to 2, from 0 to 4, or from 0 to 12, where 0 meant no signs of malocclusion or dental disease and values > 0 indicated the presence of signs in individual teeth. Next the values for incisor, premolar, and molar teeth were compared using the Kruskal–Wallis test followed by Dunn’s multiple comparisons test, as no Gaussian distributions of consecutive data series were demonstrated by the Shapiro–Wilk normality test. On the other hand, the of values for canine teeth dental diseases were compared using the Mann–Whitney test, as again, no Gaussian distributions of even one data series were demonstrated by the Shapiro–Wilk normality test. The data were presented in tables with mean with the range, and various superscripts mark statistical differences. Differences among distributions of consecutive malocclusions or dental diseases were indicated with individual *p*-values when *p* < 0.05.

## 3. Results

### 3.1. The Total Prevalence of Malocclusions and Dental Diseases

A total of 7912 teeth were examined in this study (Table 1). The specimens consisted of 2444 incisor teeth, 2462 premolar teeth, and 2471 molar teeth, as oligodontia was noted in the case of 28 incisors, 10 premolars, and 3 molars. Moreover, polydontia was observed in the case of 2 molar teeth. Canine teeth were observed in male horses only, with 512 canine teeth examined in all. Finally, a total of 23 wolf teeth were seen in 16 horses.

At least one malocclusion was found in 2336 incisor teeth from a total of 191 horses, meaning only 9 horses were free of incisor malocclusions. Malocclusion of canine teeth was found in 2 teeth in 2 different horses, thus 126 male and all female horses were free from this condition. Malocclusion of wolf teeth was present in six teeth in four horses, so two two-sided conditions were noted. Only 1 horse lacked premolar malocclusions, with at least one premolar malocclusion detected each of the remaining 205 horses, affecting a combined total of 2450 teeth. Similarly, only 1 horse was free of molar malocclusions, while 205 horses exhibited molar malocclusions in a total of 2459 teeth. At least one malocclusion was found to affect 7253 teeth, which constitutes over 91.7% of all examined teeth.

Disease was observed in 89 incisor teeth in 17 horses, while 189 horses appeared to be free of incisor diseases. Diseases of canine teeth were found in 88 teeth in 44 horses, thus 84 male and all female horses were clear. Only two diseased wolf teeth were found in different horses. Diseases of premolar and molar teeth were present in 27 and 50 teeth in 20 and 33 horses, respectively, thus 186 and 173 horses were free of these conditions. In total, at least one disease was found in 256 teeth, which constitutes only 3.2% of all examined teeth. Thus, the prevalence of malocclusions was notably different than that of dental diseases. Among the studied population, the prevalence of dental malocclusions with the exception of those affecting canine teeth was higher than the prevalence of dental diseases (*p* < 0.0001). Concerning the number of affected teeth, dental diseases most often affected incisors (34.8%) and canines (34.4%), whereas malocclusions were most commonly observed in incisors (32.2%), premolars (33.8%), and molars (33.9%) (*p* < 0.0001) (Table 1).

### 3.2. The Age-Related Prevalence of Malocclusions and Dental Diseases

In the group of horses up to 5 years old, at least 1 malocclusion was found in 143 incisor teeth from 12 horses, thus 8 horses were free of incisor malocclusions. No malocclusions of canine teeth were seen. Malocclusions of wolf teeth were noted in two teeth in one horse. Malocclusions were noted in all 240 premolar and 240 molar teeth of all 20 horses. Dental disease affected only one wolf tooth, while all remaining incisor, canine, premolar, and molar teeth were free from clinical signs of disease.

In the group of horses between 6 and 10 years of age, at least one malocclusion was found in all incisor, premolar, and molar teeth, affecting 900, 899, and 900 teeth of 20 horses, respectively. Malocclusions were noted in one canine tooth in one horse and four wolf teeth in three horses. Dental diseases were noted in five incisor, one wolf, four premolar, and five molar teeth, in four, one, three, and four horses, respectively. Dental diseases of canine teeth were observed in 28 teeth in 14 horses.

Among horses between 11 and 15 years of age, of all incisor, premolar, and molar teeth, only one incisor showed no signs of malocclusion. Malocclusion was additionally found in one canine tooth. In the case of wolf teeth, no malocclusions or disease were noted. Dental diseases were observed in 17 incisors, 10 premolars, and 14 molars, in 6, 6, and 10 horses, respectively. Canine tooth disease was again common, appearing in 34 teeth in 17 horses.

In the oldest group of horses, those over 15 years of age, not all incisors, premolars, or molars were affected by malocclusions. However, only 610, 627, and 634 teeth of the respective type were affected in the case of 53 horses. No malocclusions were noted in canine or wolf teeth, and wolf teeth were additionally free of dental disease. Dental diseases were, however, evident in 67 incisor, 26 canine, 13 premolar, and 31 molar teeth, in 7, 18, 11, and 19 horses, respectively.

In total, malocclusions and dental diseases were seen in 625 teeth and 1 tooth in the youngest group, 2704 and 43 teeth in the young group, 2053 and 75 teeth in the old group, as well as 1871 and 137 teeth in the oldest group. The prevalence of malocclusions differed significantly from the prevalence of dental diseases across all four age groups. In all age groups, the prevalence of malocclusions was higher (*p* < 0.0001) for incisor, premolar, and molar teeth. Canine teeth were affected more often by dental diseases than malocclusions in the three groups of horses over 5 years old. Wolf teeth were affected more often by malocclusions than by dental diseases in the two groups of horses up to 10 years old, while in the older horses no sign of either ailment appeared. Dental diseases were most often related to canine teeth in horses between 6–10 years old (65.1%) and those between 11–15 years old (45.3%), whereas in the oldest group of horses, diseases of the incisors were the most frequently reported (48.9%), followed by the molars (22.6%), and then the canines (19.0%). Malocclusions were most often related to the incisor, premolar, and molar teeth in all examined age groups, ranging from 22.9% to 38.4% of all affected teeth in individual groups (*p* < 0.0001) (Table 2). 

### 3.3. The Gender-Related Prevalence of Malocclusions and Dental Diseases

Among male horses, at least one malocclusion was found in 1449 incisor teeth from 122 horses, leaving only 6 male horses free of incisor malocclusion. Malocclusion of canine teeth was found in 2 teeth in 2 male horses, so the remaining 126 horses were free from this condition. Malocclusion was noted in six wolf teeth in four male horses. Only one male horse was free from malocclusions of premolar and molar teeth, with these conditions noted in 127 male horses in 1521 and 1525 teeth, respectively. Diseases of incisor teeth were observed in 45 incisors in 11 male horses, while 117 male horses remained unaffected. Diseases of canine teeth were noted in each of the previously reported horses. Disease was found in only one wolf tooth in one male horse. Diseases of premolar and molar teeth were noted in 16 and 24 teeth in 12 and 18 horses, respectively, thus 116 and 110 horses were free of cheek teeth diseases.

Among female horses, at least one malocclusion was found in 887 incisor teeth from 75 horses, so the incisors of 3 female horses were free of malocclusions. No malocclusions were found in canine or wolf teeth. All studied horses were affected with malocclusions of premolar and molar teeth, thus 929 and 934 affected teeth were noted, respectively. Dental diseases were noted in 44 incisors, 1 wolf tooth, 11 premolars, and 26 molars, in 6, 1, 8, and 16 female horses, respectively. In total, malocclusions were evident in 4503 teeth of male horses and 2750 of females, which constitutes 56.9% and 34.8% of all examined teeth. Dental diseases were noted in 174 and 80 teeth of the respective gender groups, corresponding to 2.2% and 1.0% of all examined teeth. The prevalence of malocclusions differed significantly from the prevalence of dental diseases in both male and female groups. In both gender groups, the prevalence of malocclusions was higher (*p* < 0.0001) for incisor, premolar, and molar teeth. The canine teeth in males were affected more often by dental diseases than by malocclusions, whereas in female horses, the presence of these teeth was not recorded. Concerning the distribution of the observed pathologies, dental diseases most often affected canines in male horses (50.6%) and incisors in females (53.7%), whereas malocclusions predominantly affected incisors (male: 32.2%; female: 32.3%), premolars (male: 33.8%; female: 33.8%), and molars (male: 33.9%; female: 34.0%) (*p* < 0.0001) (Table 3).

### 3.4. The Breed-Related Prevalence of Malocclusions and Dental Diseases

Among Polish warmbloods, at least one malocclusion was found in 1270 incisor teeth from 108 horses, so only 1 horse was malocclusion free. No malocclusions of canine teeth were noted, and malocclusions of wolf teeth were observed in only four teeth in two horses. Malocclusions of premolars and molars were observed in all examined horses in this group, affecting 1304 and 1309 teeth, respectively. Disease was observed in 55 incisors in 11 horses and 70 canines in 38 horses, while 98 and 35 Polish warmbloods were unaffected. Only two wolf teeth in two horses were found to be diseased. Diseases of premolars and molars were noted in the case of 17 and 26 teeth in 17 and 26 horses, respectively, thus 95 and 90 horses were free of cheek teeth diseases.

In the group of ponies, at least one malocclusion was found in all incisor, premolar, and molar teeth, affecting 466, 464, and 467 teeth, respectively, in 39 horses. Thus, for each tooth type only one horse was malocclusion free. No malocclusions of canine teeth were noted, and malocclusions of wolf teeth were observed in only one tooth in one horse. Dental diseases were noted in 18 incisors, 8 canines, 6 premolars, and 12 molars, in 2, 4, 3, and 6 horses, respectively, whereas no dental disease was observed in wolf teeth.

In the group comprising “other” horse breeds, at least one malocclusion was found in 600 incisor teeth from 50 horses, with the incisors of 7 horses appearing malocclusion free. Malocclusions were noted in two canine teeth in two horses and one wolf tooth in one horse. All horses in this group were affected with premolar and molar malocclusions, thus 682 and 683 affected teeth were noted, respectively. Dental diseases were noted in 16 incisors, 10 canines, 4 premolars, and 12 molars, in 4, 4, 3, and 8 horses, respectively. No dental disease of wolf teeth was found in this group.

In total, malocclusions and dental diseases were evident in 3887 and 170 teeth of Polish warmblood horses, 1398 and 44 teeth of ponies, and in 1968 and 42 teeth “other” breeds. The prevalence of malocclusions and dental diseases differed significantly within the three breed-based groups. In all breed-related groups, the prevalence of malocclusions was higher (*p* < 0.0001) for incisor, wolf, premolar, and molar teeth. Similarly, in all breed-related groups, the prevalence of dental diseases was higher (*p* < 0.0001) for canine teeth. Concerning the number of affected teeth, malocclusions were most often related to the incisor, premolar, and molar teeth in the group of Polish warmblood horses (32.7%, 33.5%, and 33.7%), in the group of ponies (33.3%, 33.2%, and 33.4%), as well as in the group of “other” breeds of horses (30.5%, 34.7%, and 34.7%). Dental diseases were most often related to canine teeth in the group of Polish warmblood horses (41.2%) and to incisor teeth in the groups of ponies (40.9%) and “other” breeds of horses (38.1%) (*p* < 0.0001) (Table 4).

### 3.5. The Frequency Distribution of Malocclusions and Dental Diseases of the Incisor Teeth

**Figure 3:**
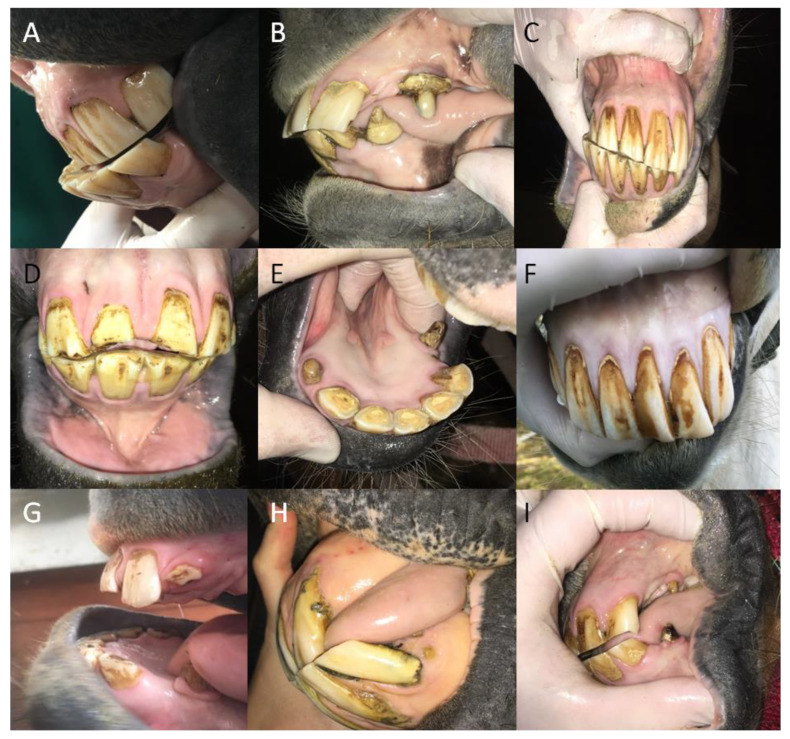
The samples of the malocclusions of the incisor teeth in horses. Underbite (**A**); overbite (**B**); curvature (**C**); diastema (**D**); oligodontia (**E**); polydontia (**F**); retained deciduous teeth (RDT) (**G**); the Equine Odontoclastic Tooth Resorption and Hypercementosis (EOTRH) syndrome (**H**); and hooks (**I**).

The curvature occurred with a significantly higher frequency than did other malocclusions of the incisor teeth, regardless of the studied group (*p* < 0.0001). One may observe that in all incisor teeth no polydontia appeared. Moreover, no overbite, diastema, EOTRH, or hooks were found in the youngest group of horses, those up to 5 years old. In the group of young horses between 6 and 10 years of age, no oligodontia or EOTRH were noted. In the groups of horses between 11–15 years old and those over 15 years of age, no underbite, diastema, or RDT were observed. In the gender-based groups, while polydontia was absent among both males and females, the female group also lacked signs of diastema. Similarly, in the breed-based groups, no diastema was noted in Polish warmblood horses. Among the ponies, no hooks were observed, while in the group of “other” breeds, no underbite, diastema, oligodontia, or RDT were recognized. No significant differences were found among the distributions of the remaining malocclusions (Table 5; Figure 3).

**Figure 4:**
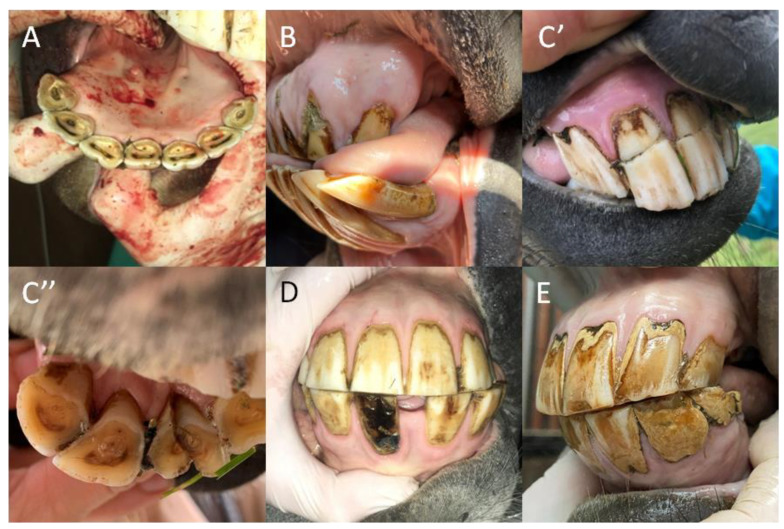
The samples of the dental diseases of the incisor teeth in horses. Supernumerary (**A**); loose teeth (**B**); fractures ((**C**): transverse fracture, (**C’**); sagittal fracture, (**C”**)); caries (**D**); and calculus (**E**).

Calculus occurred with a significantly higher frequency than other dental diseases of the incisor teeth in the groups of all studied horses (*p =* 0.0007), the oldest horses (*p =* 0.0005), the male horses (*p =* 0.021), and the Polish warmblood horses (*p =* 0.009), but not in the remaining sub-groups. No supernumerary incisor teeth appeared in any group. Moreover, no dental diseases were found in the group of the youngest horses up to 5 years old. No loose teeth were noted in groups of horses between 6–10 years old or 11–15 years old, or in male horses, ponies, or “other” breeds. Additionally, no fractures were found in groups of horses over 15 years old, in ponies, or in “other” breeds. Finally, no caries was observed in horses between 11–15 years old or older in female horses, Polish warmbloods, or ponies. No significant differences were found among the distributions of the remaining malocclusions (Table 6; Figure 4).

### 3.6. The Frequency Distribution of Malocclusions and Dental Diseases of Canine and Wolf Teeth

**Figure 5:**
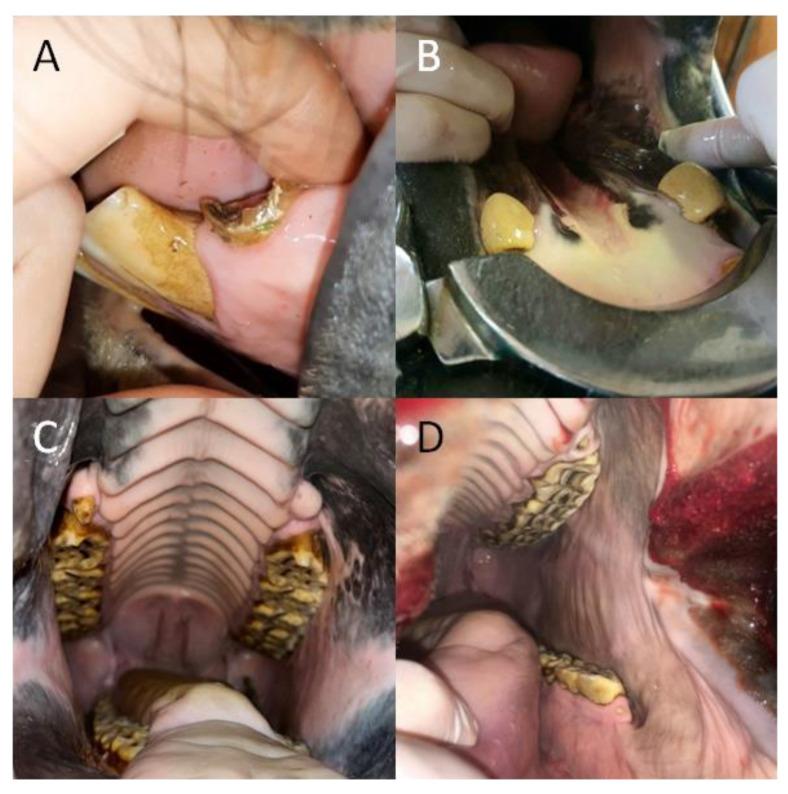
The samples of the malocclusions and dental diseases of the canine and wolf teeth in horses. For the canine teeth: fractures (**A**) and calculus (**B**). For the wolf teeth: blind wolf tooth (**C**) and fractures (**D**).

Regarding malocclusions, non-erupted canines occurred in groups of horses between 6 and 10 years old, and 11 and 15 years old, in male horses, and in “other” breeds, whereas blind wolf teeth occurred in groups of horses 0–5 years old, between 6–10 years-old, in male horses, and in all studied breeds. With respect to dental diseases, fractures of canine teeth did not appear in horses 0–5 years of age or 6–10 years of age in either gender group, or among Polish warmbloods. Moreover, calculus of the canines did not occur in the youngest group up to 5 years old or in female horses (in whom canines were absent altogether). In all other groups, calculus appeared more frequently than fractures (Table 7; Figure 5).

### 3.7. The Frequency Distribution of Malocclusions and Dental Diseases of Cheek Teeth

**Figure 6:**
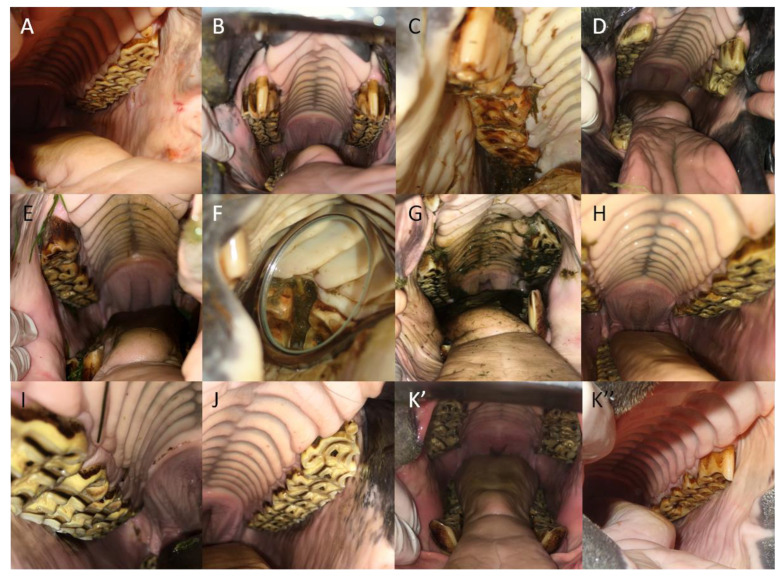
The samples of the malocclusions of the premolar and molar teeth in horses. Sharp enamel points (**A**); overgrown tooth (**B**); wave mouth (**C**); step mouth (**D**); displaced teeth (**E**); diastema (**F**); oligodontia (**G**); polydontia (**H**); retained deciduous teeth (RDT) (**I**); excessive transverse ridges (ETR) (**J**); and hooks (**K’**,**K”**).

Sharp enamel points occurred with a significantly higher frequency than other malocclusions of the premolar and molar teeth, irrespective of the studied group (*p* < 0.0001). No polydontia of the premolars appeared in any group, and no molars affected by RDT occurred. The remaining malocclusions occurred in both premolar and molar teeth with varying frequency among for the studied groups. In the youngest horses up to 5 years old, overgrown and displaced teeth, RDT, and ETR were found to affect premolar teeth, while only ETR and hooks appeared to affect molars. Regarding premolar teeth, the same malocclusions (overgrown tooth, wave mouth, diastema, ETR, and hooks) were noted in groups of horses 6–10 and 11–15 years old, except for oligodontia, which only appeared in the group of 6–10 years old (Table 8; Figure 6).

Similarly, regarding molar teeth, in groups of horses between 6–10 and 11–15 years old, the same malocclusions (overgrown teeth, wave mouth, displaced teeth, diastema, ETR, and hooks) were observed, with the exception of polydontia, which only appeared in horses 11–15 years old. In the group of the oldest horses, those over 15 years of age, all malocclusions were recognized, except for premolar polydontia and RDT and molar RDT. In the gender-based groups, in addition to the lack of either premolar polydontia or molar RDT, there were no signs of premolar step mouth or RDT females. Moreover, no signs of molar oligodontia were seen in males or molar step mouth in females. Similarly, in the breed-related groups, except for lack of premolar polydontia and molar RDT, all other premolar and molar malocclusions were noted among Polish warmbloods. Among the ponies, no premolar step mouth, displaced teeth, RDT, or ETR were observed, and none had molar step mouth, polydontia, or ETR. Finally, in the group of “other” breeds, no premolar or molar step mouth, polydontia, or RDT were recognized (Table 9).

**Figure 7:**
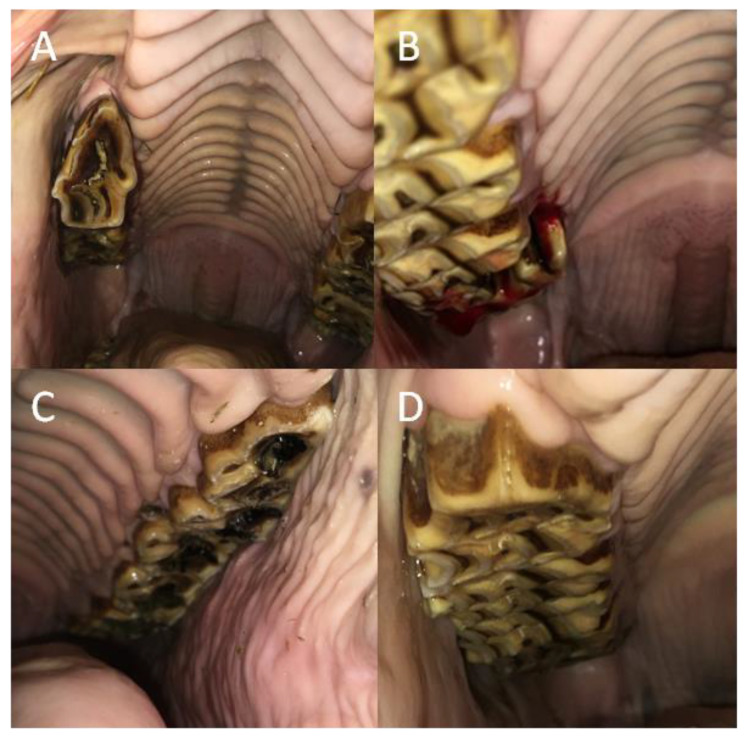
The samples of the dental diseases of the premolar and molar teeth in horses. Loose teeth (**A**); fractures (**B**); caries (**C**); and calculus (**D**).

In all studied horses, dental diseases occurred with the same frequency distribution in premolar teeth (*p =* 0.059), whereas in molar teeth, caries occurred with a significantly higher frequency than loose teeth or calculus (*p* < 0.0001). Concerning premolars among sub-groups, caries was noted with a higher frequency than other dental diseases in horses 6–10 years old (*p =* 0.028), as well as with a higher frequency than calculus in horses over 15 years of age (*p =* 0.036). Concerning molar teeth among the sub-groups, caries was recognized with a higher frequency than other dental diseases in horses 11–15 years old (*p =* 0.004), in male horses (*p =* 0.002), and in Polish warmbloods (*p* < 0.0001), as well as with a higher frequency than calculus in horses over 15 years of age (*p =* 0.003) and in female horses (*p =* 0.028). In the case of cheek teeth, no calculus was observed. Moreover, no dental diseases were found in the cheek teeth of horses up to 5 years old. In addition to a lack of calculus, no loose premolars were noted in horses 6–10 years old or 11–15 years old. Similarly, no loose molars were observed in the horses from the “other” breeds group. Additionally, no premolar fractures were recognized in horses 6–10 years old, in ponies, or in the “other” breeds (Table 10; Figure 7).

## 4. Discussion

To the best of our knowledge, the current paper presents for the first time a descriptive study of the prevalence and frequency distribution of malocclusions and dental diseases of all types of teeth in a select population of horses housed in the Mazovia region of Poland. As horse populations differ among countries and regions, a specific characterization of dental disorder occurrence may be helpful for local equine practitioners. In the current study, 31% of examined horses presented with dental disorders in the area of the incisor teeth, which is less than the previously reported 53% of Polish horses [16]. No data regarding the prevalence of dental disorders of the cheek teeth have been previously reported in Poland. However, 70% of examined horses in Canada [12] and 87% in Australia [15] demonstrated dental disorders in the area of the cheek, which is more than the 62% reported herein. In the current study, dental disorders occurred in at least one tooth in 95% of examined horses, which is close to the prevalence reported in Australia (94%) [15] and Scotland (87%) [14], and higher than the prevalence reported in the USA (80%) [11] and UK (from 79% [3] to 42% [13]). Given the variability in the reported prevalence in a single geographic location such as the UK, other factors such as the dental examination protocol, equipment, and type of study design employed should be taken into consideration as the cause of variability in the incidence of malocclusions and dental disease.

Regardless of the study group, malocclusions of incisor, premolar, and molar teeth occurred with a higher prevalence than did dental diseases. As previously referenced authors [3,11,13,14,15,16] did not differentiate dental problems into malocclusions and dental diseases, the findings of the current study provide valuable clinical evidence justifying the need for periodic dental examination of horses. In many cases, malocclusions can be corrected using simple procedures, and this can prevent the development and consequences of very advanced disorders including dental diseases [43]. Some congenital disorders, such as underbite and overbite, which are easily diagnosed in young foals, can be corrected early [43,44], preventing the development of further dental disorders. Moreover, the event of underbite or overbite or conformational variations in the cheek teeth may also occur, which may result in the possible formation of hooks of the maxillar or mandibular teeth [31]. This is consistent with the current observation that no dental diseases were noted in the youngest age group. Notably, the prevalence of dental diseases, but not malocclusions, increased with age. Given that some dental disorders, especially dental diseases, are common to geriatric horses, educating older horse owners and recommending prophylactic dental treatments for this age group are key to minimizing the impact of debilitating dental disease [45]. Interestingly, the prevalence of incisor and cheek teeth malocclusions was similar across divisions in both gender-based and in breed-based groups when the percentage of horses was considered in relation to the number of affected teeth in each group. In contrast, the prevalence of both malocclusions and dental diseases of canine and wolf teeth was higher in the male than female group, as no canine teeth were present in mares [39].

Regarding the distribution of specific malocclusions for each tooth, curvatures most frequently affected incisor teeth while sharp enamel points plagued cheek teeth. In the case of incisor teeth, the line created by the occlusal surfaces between the maxillary and mandibular incisors should lie on the horizontal plane, and any deviation thereof, be it ventral, dorsal, diagonal, or irregular, constitutes an abnormality [46]. In the current study, ventral curvature was found in 157 horses. Irregular curvature and diagonal bite were found in 21 and 10 horses respectively, whereas dorsal curvature was not observed. Depending on recent reports, ventral curvature may be considered an acquired, age-related disorder, probably secondary to cheek teeth disorders [46] or correct conformation [9]. However, regardless of this characterization, the gradual correction of ventral curvature is recommended to restore the correct conformation of the incisors, thus avoiding the development of an abnormal occlusal surface [46], which may affect the horse’s chewing ability [47]. In the current study, sharp edges were found in 204 horses on the buccal surface of the maxillary cheek teeth and the lingual surface of the mandibular cheek teeth, posing a risk to the cheek or tongue mucosa, respectively. The extent of the mucosal damage at the site of irritation may involve its partial destruction (i.e., erosion) or damage to the full thickness of the mucosa (i.e., ulceration). In extreme cases, laceration may occur, involving complete rupture of the mucosa over a considerable length. As a result of long-term irritation to the mucosa, buccal calluses may form. In such cases, there is loss of superficial epithelium together with necrotic signs or areas of excessive mucosal epithelial growth [7]. The anatomical arrangement of the teeth in the lower and upper dental arches is such that the curvature of the upper arch does not fully match that of the lower arch, the lower arch is straighter, and the distance between the right and the left arch is smaller in the mandible in comparison to the maxilla [48]. Furthermore, the cheek teeth of the mandible are physiologically narrower than their antagonists in the maxilla. This arrangement of the teeth and their anatomical dependence on each other causes uneven abrasion of the occlusal surfaces, predisposing to the formation of sharp points on the buccal edges of the maxillary cheek teeth and the lingual edges of the mandibular cheek teeth, which in turn leads to mechanical damage to the cheek mucosa or the tongue [49,50].

With respect to the distribution of specific dental diseases for each tooth, calculus was most frequently observed on incisor teeth and caries in cheek teeth. Calculus, the accumulation of tartar, occurs as a result of the mineralization of the bacterial plaque flora on the teeth [51]. The canine and incisor teeth of the mandible are most frequently predisposed to calculus deposition [51], which is in line with the observations in the present study. On the other hand, maxillary cheek teeth are most frequently predisposed to caries, as each maxillary cheek tooth contains two semicircular funnel-like infundibula filled with cementum [51]. In the current study, infundibular caries was found in 26 horses, most often in the first molars. However, the grading of caries was not investigated in this study. Clinically, a 4-grade scale of infundibular caries advancement is used to describe the process and the structures involved. In grade 1, only the cementum is affected. In grade 2, the cementum and adjacent enamel are affected. In grade 3, the cementum, enamel, and dentin are affected. In grade 4, the integrity of the tooth is affected [46]. In many cases, the presence of infundibular caries mechanically weakens the clinical crown of the cheek tooth and thus predisposes to increased wear. Consequently, the opposing mandibular tooth tends to protrude excessively [47]. Advanced infundibular caries of the maxillary cheek teeth often predisposes to midline sagittal fractures and apical infection [52,53].

Interestingly, the frequency distribution of a few dental disorders seems to be age-related, rather than gender- or breed-related. The exceptions were non-erupted canines, blind wolf teeth, fractures of canine and wolf teeth, and calculus of canine teeth, which, due to the gender-dependent occurrence of canine and to some extent wolf teeth [39], were found in males and not in females. In the case of incisor teeth, EOTRH and calculus did not appear in horses up to 5 years old but appeared frequently in horses over 15 years of age. The current observations are in line with the previous study on age predilection in the occurrence of both malocclusion [54] and disease [51]. The age-related risk of EOTRH occurrence was confirmed in a subsequent study [34,55]. In the case of cheek teeth, wave mouth, step mouth, fractures, and caries were absent in horses up to 5 years of age but appeared frequently in horses over 15 years old. The etiology of wave mouth is unclear, but an age-related component has been postulated [14]. The eruption of a horse’s cheek teeth depends on the continuous relaxation and contraction of the periodontal fibers. During periodontal disease and aging, subsequent fiber loss may delay the eruption of affected teeth and thus lead to the formation of a wave mouth [14]. On the other hand, a step mouth is an abnormal bite, which most often appears as a result of the overgrowth of a cheek tooth due to the lack or fracture of its antagonist [56,57]. Therefore, an increased occurrence of check teeth fractures may predispose to step mouth [14,58], which is in keeping with the current observations.

### Limitations

In this study, the reader’s attention should be paid to several limitations that should be taken into account when assessing the value of the presented results. As indicated at the beginning of the discussion section, the horse populations as well as the occurrence of dental disorders differ among countries and regions [3,11,12,13,14,15,16]. One should note that this paper presents a descriptive study of the prevalence and frequency distribution of dental disorders in a select population of horses housed in the Mazovia region of Poland. Thus, the presented results are of much greater local importance, especially for local equine practitioners, than of general relevance.

Moreover, this study was conducted on a specific and restricted population, not on a region-wide reference population of local horses as assumed in population screening. In recent studies, two methods have been used to obtain data on the occurrence of dental disorder in horse populations. The first approach involves screening horses’ teeth at slaughter [5,6], while the second involves screening horses’ teeth during annual routine examination of the oral cavity [2,3,4]. Post mortem examinations of equine heads obtained from slaughterhouses [5,6,8,9,10] evaluate sample groups that may be considered representative of the larger study population, whereas routine examinations of live horses [2,3,4,7] satisfy the criteria of descriptive studies. Each method of study group selections has its own limitations. The use of horse heads obtained from slaughterhouses is constrained by the limited availability of sensitive data. Slaughterhouses in Poland are not legally permitted to provide private information, even for scientific purposes, regarding the origin, pedigree, age, or reason for slaughter of horses. Therefore, even though the group of slaughtered horses is more representative of the local population, legal restrictions significantly impede any effort to classify data according to age, gender, or breed. On the other hand, the use of live horses presented for dental examinations by consenting owners provides access to a complete set of sensitive data. However, such studies may nonetheless be limited by inferior representation, as horses presented for dental examinations may, for various reasons, not exhibit the same prevalence of dental disease as those not included in such a study. Therefore, for the purpose of the current study, routine inspection during the annual dental check-up was performed in all horses housed in 45 private stables, 1% of all small horse farms (4550) registered in the Mazovian Voivodeship [18], regardless of their oral health status [7]. Moreover, demographic data were provided to substantiate the representative nature of the sample group. Among 37,397 horses registered in the Mazovian Voivodeship [18], approximately 15% (5550 horses) are housed in smaller-scale (111) equestrian centers [19], whereas about 85% (31,847 horses) are kept in (4550) private small horse farms [18]. The annual routine dental examination on small horse farms is generally easier to perform due to the smaller number of horses housed in a single stable (an average of 4–6 horses per stable in this study) compared with large equestrian centers (averaging 50 horses per stable). Furthermore, private owners place greater emphasis on the welfare of their horses and are willing to spend more time on routine inspection during an annual dental check-up, regardless of oral health status. Thus, the observations described below should be considered with respect to the selected group of privately owned horses housed in the Mazovia region of Poland.

## 5. Conclusions

In a select group of privately owned horses housed in the Mazovia region of Poland, 95% demonstrated dental disorders, with a similar percentage of pathology observed in the incisor teeth (31%) and the cheek teeth (31% each for premolars and molars). Irrespective of age, gender, and breed groupings, malocclusions of incisor, premolar, and molar teeth occurred with a higher prevalence than did dental diseases. Curvatures and sharp enamel points were the most frequently occurring malocclusions of the incisor and cheek teeth, respectively, while calculus and caries were the most frequently distributed dental diseases of the incisor and cheek teeth, respectively.

## Figures and Tables

**Table 1:** The prevalence of malocclusions and dental diseases in 7912 examined teeth of 206 horses.

Teeth	No. of Teeth	Teeth with Malocclusions	Teeth with Dental Diseases
Incisor	2444 (30.9)	2336 (29.5; 32.2)	89 (1.1; 34.8)
Canine	512 (6.5)	2 (0.03; 0.03)	88 (1.1; 34.4)
Wolf	23 (0.3)	6 (0.1; 0.1)	2 (0.03; 0.8)
Premolar	2462 (31.1)	2450 (31.0; 33.8)	27 (0.3; 10.5)
Molar	2471 (31.2)	2459 (31.1; 33.9)	50 (0.6; 19.5)
Total	7912	7253 (91.7; 100.0)	256 (3.2; 100.0)
Chi-square test		*p* < 0.0001; *p* < 0.0001	

Data are presented as n (% of all horses supported with *p* and *%* of horses in relation to the number of affected teeth supported with *p*). Differences were considered significant when *p* < 0.05.

**Table 2:** The prevalence of malocclusions and dental diseases in 7912 examined teeth of 206 horses by horses’ age (*n* = 20, 0–5 years; *n* = 75, 6–10 years; *n* = 57, 11–15 years; *n* = 54, > 15 years).

Age	Teeth	No. of Teeth	Teeth with Malocclusions	Teeth with Dental Diseases
0–5 years	Incisor	239 (3.0)	143 (1.8; 22.9)	0 (0; 0)
	Canine	60 (0.8)	0 (0; 0)	0 (0; 0)
	Wolf	5 (0.1)	2 (0.03; 0.3)	1 (0.01; 100.0)
	Premolar	240 (3.0)	240 (3.0; 38.4)	0 (0; 0)
	Molar	240 (3.0)	240 (3.0; 38.4)	0 (0; 0)
Total		784 (9.9)	625 (7.9)	1 (0.01)
Chi-square test		*p* < 0.0001; *p* < 0.0001		
6–10 years	Incisor	900 (11.4)	900 (11.4; 33.3)	5 (0.1; 11.6)
	Canine	196 (2.5)	1 (0.01; 0.04)	28 (0.35; 65.1)
	Wolf	12 (0.2)	4 (0.1; 0.1)	1 (0.01; 2.3)
	Premolar	899 (11.4)	899 (11.4; 33.2)	4 (0.05; 9.3)
	Molar	900 (11.4)	900 (11.4; 33.3)	5 (0.1; 11.6)
Total		2907 (36.7)	2704 (34.2)	43 (0.5)
Chi-square test		*p* < 0.0001; *p* < 0.0001		
11–15 years	Incisor	684 (8.6)	683 (8.6; 33.3)	17 (0.2; 22.7)
	Canine	132 (1.7)	1 (0.01; 0.05)	34 (0.4; 45.3)
	Wolf	1 (0.01)	0 (0; 0)	0 (0; 0)
	Premolar	684 (8.6)	684 (8.6; 33.3)	10 (0.1; 13.3)
	Molar	685 (8.7)	685 (8.7; 33.4)	14 (0.2; 18.7)
Total		2185 (26.7)	2053 (25.9)	75 (0.9)
Chi-square test		*p* < 0.0001; *p* < 0.0001		
>15 years	Incisor	622 (7.9)	610 (7.7; 32.6)	67 (0.9; 48.9)
	Canine	124 (1.6)	0 (0; 0)	26 (0.3; 19.0)
	Wolf	5 (0.1)	0 (0; 0)	0 (0; 0)
	Premolar	639 (8.1)	627 (7.9; 33.5)	13 (0.2; 9.5)
	Molar	646 (8.2)	634 (8.0; 33.9)	31 (0.4; 22.6)
Total		2036 (25.7)	1871 (23.6)	137 (1.7)
Chi-square test		*p* < 0.0001; *p* < 0.0001		
Total		7912	7253 (91.7)	256 (3.2)

Data are presented as n (% of all horses supported with *p* and *%* of horses in relation to the number of affected teeth supported with *p*). Differences were considered significant when *p* < 0.05.

**Table 3:** The prevalence of malocclusions and dental diseases in 7912 examined teeth of 206 horses by horses’ gender (*n* = 128, male; *n* = 78, female).

Gender	Teeth	No. of Teeth	Teeth with Malocclusions	Teeth with Dental Diseases
Male	Incisor	1521 (19.2)	1449 (18.3; 32.2)	45 (0.6; 25.9)
	Canine	512 (6.5)	2 (0.03; 0.04)	88 (1.1; 50.6)
	Wolf	14 (0.2)	6 (0.1; 0.1)	1 (0.01; 0.6)
	Premolar	1533 (19.4)	1521 (19.2; 33.8)	16 (0.2; 9.2)
	Molar	1537 (19.4)	1525 (19.3; 33.9)	24 (0.3; 13.8)
Total		5117 (64.7)	4503 (56.9)	174 (2.2)
Chi-square test		*p* < 0.0001; *p* < 0.0001		
Female	Incisor	923 (11.7)	887 (11.2; 32.3)	44 (0.6; 53.7)
	Canine	0 (0)	0 (0; 0)	0 (0; 0)
	Wolf	9 (0.1)	0 (0; 0)	1 (0.01; 1.2)
	Premolar	929 (11.7)	929 (11.7; 33.8)	11 (0.1; 13.4)
	Molar	934 (11.8)	934 (11.8; 34.0)	26 (0.3; 31.7)
Total		2795 (35.3)	2750 (34.8)	82 (1.0)
Chi-square test		*p* < 0.0001; *p* < 0.0001		
Total		7912	7253 (91.7)	256 (3.2)

Data are presented as n (% of all horses supported with *p* and *%* of horses in relation to the number of affected teeth supported with *p*). Differences were considered significant when *p* < 0.05.

**Table 4:** The prevalence of malocclusions and dental diseases in 7912 examined teeth of 206 horses by horses’ breed (*n* = 109, Polish warmblood; *n* = 40, pony; *n* = 57, “other”).

Breed	Teeth	No. of Teeth	Teeth with Malocclusions	Teeth with Dental Diseases
Polish warmblood	Incisor	1282 (16.2)	1270 (16.1; 32.7)	55 (0.7; 32.4)
	Canine	292 (3.7)	0 (0; 0)	70 (0.9; 41.2)
	Wolf	13 (0.2)	4 (0.1; 0.1)	2 (0.03; 1.2)
	Premolar	1304 (16.5)	1304 (16.5; 33.5)	17 (0.2; 10.0)
	Molar	1309 (16.5)	1309 (16.5; 33.7)	26 (0.3; 15.3)
Total		4200 (53.1)	3887 (49.1)	170 (2.1)
Chi-square test		*p* < 0.0001; *p* < 0.0001		
Pony	Incisor	478 (6.0)	466 (5.9; 33.3)	18 (0.2; 40.9)
	Canine	100 (1.3)	0 (0; 0)	8 (0.1; 18.2)
	Wolf	5 (0.1)	1 (0.01; 0.1)	0 (0; 0)
	Premolar	476 (6.0)	464 (5.9; 33.2)	6 (0.1; 13.6)
	Molar	479 (6.1)	467 (5.9; 33.4)	12 (0.2; 27.3)
Total		1538 (19.4)	1398 (17.7)	1494 (18.9)
Chi-square test		*p* < 0.0001; *p* < 0.0001		
“Other”	Incisor	684 (8.6)	600 (7.6; 30.5)	16 (0.2; 38.1)
	Canine	120 (1.5)	2 (0.01; 0.1)	10 (0.1; 23.8)
	Wolf	5 (0.1)	1 (0.01; 0.1)	0 (0; 0)
	Premolar	682 (8.6)	682 (8.6; 34.7)	4 (0.05; 9.5)
	Molar	683 (8.6)	683 (8.6; 34.7)	12 (0.2; 28.6)
Total		2174 (27.5)	1968 (24.9)	42 (0.5)
Chi-square test		*p* < 0.0001; *p* < 0.0001		
Total		7912	7253 (91.7)	256 (3.2)

Data are presented as n (% of all horses supported with *p* and *%* of horses in relation to the number of affected teeth supported with *p*). Differences were considered significant when *p* < 0.05.

**Table 5:** The frequency distribution of malocclusions of incisor teeth in the total 7912 examined teeth of 206 horses.

	Underbite	Overbite	Curvature	Diastema	Oligodontia	Polydontia	RDT	EOTRH	Hooks	*p*
Total	0.12 (12) a	1.17 (12) a	11.29 (12) b	0.01 (2) a	0.14 (12) a	0 (0) a	0.01 (1) a	0.87 (12) a	0.02 (2) a	<0.0001
0–5 years	0.60 (12) a	0 (0) a	7.15 (12) b	0 (0) a	0.05 (1) a	0 (0) a	0.05 (1) a	0 (0) a	0 (0) a	<0.0001
6–10 years	0.16 (12) a	1.76 (12) a	12.00 (12) b	0.01 (1) a	0 (0) a	0 (0) a	0.01 (1) a	0 (0) a	0.01(1) a	<0.0001
11–15 years	0 (0) a	1.05 (12) a	12.00 (12) b	0 (0) a	0.02 (1) a	0 (0) a	0 (0) a	0.21 (12) a	0.04(2) a	<0.0001
>15 years	0 (0) a	0.89 (12) a	11.09 (12) b	0 (0) a	0.48 (12) a	0 (0) a	0 (0) a	3.11 (12) a	0.04 (2) a	<0.0001
Male	0.09 (12) a	1.41 (12) a	11.33 (12) b	0.01 (1) a	0.12 (12) a	0 (0) a	0.02 (1) a	1.03 (12) a	0.02 (2) a	<0.0001
Female	0.15 (12) a	0.77 (12) a	11.23 (12) b	0 (0) a	0.17 (12) a	0 (0) a	0 (0) a	0.62 (12) a	0.03 (2) a	<0.0001
PWB	0.11 (12) a	1.21 (12) a	11.55 (12) b	0 (0) a	0.24 (12) a	0 (0) a	0.01 (12) a	1.21 (1) a	0.03(2) a	<0.0001
Pony	0.30 (12) a	0.90 (12) a	11.68 (12) b	0.03 (1) a	0.05 (1) a	0 (0) a	0.03 (1) a	0.60 (12) a	0 (0) a	<0.0001
“Other”	0 (0) a	1.26 (12) a	10.53 (12) b	0 (0) a	0 (0) a	0 (0) a	0 (0) a	0.42 (12) a	0.04 (2) a	<0.0001

Data are presented as mean (with the range). Different letters in consecutive cells were statistically different when *p* < 0.05. PWB—Polish warmblood; RDT—retained deciduous teeth; EOTRH—Equine Odontoclastic Tooth Resorption and Hypercementosis syndrome.

**Table 6:** The frequency distribution of dental diseases of incisor teeth in the total 7912 examined teeth of 206 horses.

	Supernumerary	Loose Teeth	Fractures	Caries	Calculus	*p*
Total	0 (0) a	0.001 (1) a	0.02 (1) a	0.01 (1) a	0.40 (12) b	0.0007
0–5 years	0 (0)	0 (0)	0 (0)	0 (0)	0 (0)	nc
6–10 years	0 (0) a	0 (0) a	0.01 (1) a	0.03 (1) a	0.03 (2) a	0.476
11–15 years	0 (0) a	0 (0) a	0.05 (1) a	0 (0) a	0.25 (6) a	0.057
>15 years	0 (0) a	0.02 (1) a	0 (0) a	0 (0) a	1.22 (12) b	0.0005
Male	0 (0) a	0 (0) a	0.02 (1) ab	0.02 (1) ab	0.31 (12) b	0.021
Female	0 (0) a	0.01 (1) a	0.01 (1) a	0 (0) a	0.54 (12) a	0.056
PWB	0 (0) a	0.01 (1) ab	0.01 (1) ab	0 (0) a	0.46 (12) b	0.009
Pony	0 (0) a	0 (0) a	0 (0) a	0 (0) a	0.45 (12) a	0.090
“Other”	0 (0) a	0 (0) a	0 (0) a	0.04 (1) a	0.25 (12) a	0.194

Data are presented as mean (with the range). Different letters in consecutive cells were statistically different when *p* < 0.05; nc—not calculable; PWB—Polish warmblood.

**Table 7:** The frequency distribution of malocclusions and dental diseases of canine * and wolf ** teeth in the total 7912 examined teeth of 206 horses.

	Non-Erupted Canines *	Blind Wolf Tooth **	Fractures *	Calculus *	*p*	Fractures **
Total	0.01 (1)	0.03 (2)	0 (0) a	0.43 (2) b	<0.0001	0.01 (1)
0–5 years	0 (0)	0.10 (2)	0 (0)	0 (0)	nc	0.05 (1)
6–10 years	0.01 (1)	0.05 (2)	0 (0) a	0.37 (2) b	<0.0001	0.01 (1)
11–15 years	0.02 (1)	0 (0)	0 (0) a	0.60 (2) b	<0.0001	0 (0)
>15 years	0 (0)	0 (0)	0 (0) a	0.48 (2) b	<0.0001	0 (0)
Male	0.02 (1)	0.05 (2)	0 (0) a	0.69 (2) b	<0.0001	0.01 (1)
Female	0 (0)	0 (0)	0 (0)	0 (0)	nc	0.02 (1)
PWB	0 (0)	0.04 (2)	0 (0) a	0.64 (2) b	<0.0001	0.02 (1)
Pony	0 (0)	0.03 (1)	0 (0) a	0.20 (2) a	0.116	0 (0)
“Other”	0.04 (1)	0.02 (1)	0 (0) a	0.17 (2) a	0.057	0 (0)

Data are presented as mean (with the range). Different letters in consecutive cells were statistically different when *p* < 0.05; nc—not calculable; PWB—Polish warmblood.

**Table 8:** The frequency distribution of malocclusions of premolar teeth in the total 7912 examined teeth of 206 horses.

	Sharp e.	Overgrown t.	Wave m.	Step m.	Displaced t.	Diastema	Oligodontia	Polydontia	RDT	ETR	Hooks	*p*
Total	11.90(12) a	0.68 (12) b	0.52 (12) c	0.06 (12) c	0.02 (2) c	0.15 (2) bc	0.05 (4) c	0 (0) c	0.01(2) c	0.58 (12) bc	0.19 (2) b	<0.0001
0–5 years	12.00 (12) a	0.30 (2) b	0 (0) b	0 (0) b	0.20(2) b	0 (0) b	0 (0) b	0 (0) b	0.10(2) b	0.60(12) b	0 (0) b	<0.0001
6–10 years	12.00 (12) a	0.53 (4) b	0.31 (12) c	0 (0) c	0 (0) c	0.08 (2) bc	0.01(1) c	0 (0) c	0 (0) c	0.96 (12) bc	0.20 (2) bc	<0.0001
11–15 years	12.00 (12) a	0.93 (12) b	0.21(12) c	0 (0) c	0 (0) c	0.09 (2) c	0 (0) c	0 (0) c	0 (0) c	0.21(12) c	0.21(2) c	<0.0001
>15 years	11.61 (12) a	0.78 (4) b	1.33 (12) bc	0.22 (12) c	0.02 (1) c	0.35 (2) bc	0.17 (4) bc	0 (0) c	0 (0) c	0.44 (12) bc	0.22 (2) bc	<0.0001
Male	11.89 (12) a	0.63 (4) b	0.37 (12) c	0.09 (12) c	0.03 (2) c	0.13 (2) c	0.02 (1) c	0 (0) c	0.02 (2) c	0.56 (12) c	0.18 (2) c	<0.0001
Female	11.91 (12) a	0.77 (12) b	0.77 (12) bc	0 (0) c	0.01 (1) c	0.18 (2) bc	0.09 (4) c	0 (0) c	0 (0) c	0.62(12) c	0.21 (2) bc	<0.0001
PWB	11.98 (12) a	0.68(4) b	0.55 (12) c	0.09 (12) c	0.02 (2) c	0.18 (2) c	0.03 (1) c	0 (0) c	0.03 (2) c	0.38 (12) c	0.19 (2) c	<0.0001
Pony	11.60 (12) a	0.73 (4) b	0.30 (12) c	0 (0) c	0 (0) c	0.05 (1) c	0.10 (4) c	0 (0) c	0 (0) c	0 (0) c	0.13 (2) bc	<0.0001
“Other”	11.96 (12) a	0.68 (12) b	0.63 (12) b	0 (0) b	0.05 (2) b	0.09 (2) b	0.04 (2) b	0 (0) b	0 (0) b	1.47 (12) b	0.18 (2) b	<0.0001

Data are presented as mean (with the range). Different letters in consecutive cells were statistically different when *p* < 0.05. PWB—Polish warmblood; sharp e.—sharp enamel points; overgrown t.—overgrown tooth; wave m.—wave mouth; step m.—step mouth; displaced t.—displaced teeth; RDT—retained deciduous teeth; ETR—excessive transverse ridges.

**Table 9:** The frequency distribution of malocclusions of molar teeth in the total 7912 examined teeth of 206 horses.

	Sharp e.	Overgrown t.	Wave m.	Step m.	Displaced t.	Diastema	Oligodontia	Polydontia	RDT	ETR	Hooks	*p*
Total	11.93 (12) a	0.19 (4) b	0.53 (12) b	0.06 (12) b	0.03 (2) b	0.05 (2) b	0.01 (1) b	0.01(1) b	0(0) b	0.58(12) b	0.32(2) b	<0.0001
0–5 years	12 (12) a	0 (0) b	0 (0) b	0 (0) b	0 (0) b	0 (0) b	0 (0) b	0 (0) b	0 (0) b	0.60 (12) b	0.10 (2) b	<0.0001
6–10 years	12.00 (12) a	0.11 (2) b	0.32 (12) b	0 (0) b	0.01 (1) b	0.01 (1) b	0 (0) b	0 (0) b	0 (0) b	0.96 (12) b	0.28(2) b	<0.0001
11–15 years	12.00 (12) a	0.21 (2) b	0.21 (12) b	0 (0) b	0.09 (2) b	0.04 (2) b	0 (0) b	0.02 (1) b	0 (0) b	0.21 (12) b	0.21 (2) b	<0.0001
>15 years	11.72 (12) a	0.35 (4) b	1.37 (12) b	0.23 (12) b	0.02 (1) b	0.13 (2) b	0.06 (1) b	0.02 (1) b	0 (0) b	0.44 (12) bc	0.56 (2) c	<0.0001
Male	11.91 (12) a	0.23 (4) b	0.38 (12) b	0.09 (12) b	0.03 (2) b	0.05 (2) b	0 (0) b	0.01 (1) b	0 (0) b	0.56 (12) b	0.29 (2) b	<0.0001
Female	11.96 (12) a	0.12 (2) b	0.79 (12) b	0 (0) b	0.04 (2) b	0.05 (2) b	0.04 (1) b	0.01 (1) b	0(0) b	0.62 (12) b	0.36 (2) b	<0.0001
PWB	11.99 (12) a	0.24 (4) bc	0.57 (12) bc	0.11 (12) b	0.02 (2) b	0.06 (2) bc	0.01 (1) b	0.02 (1) b	0(0) b	0.33 (12) bc	0.33 (2) c	<0.0001
Pony	11.68 (12) a	0.13 (2) b	0.30 (12) b	0(0) b	0.10 (2) b	0.05 (1) b	0.03 (1) b	0(0) b	0(0) b	0(0) b	0.18 (2) b	<0.0001
“Other”	11.98 (12) a	0.14 (2) b	0.63 (12) b	0(0) b	0.02 (1) b	0.02 (1) b	0.02 (1) b	0(0) b	0(0) b	1.47 (12) b	0.39 (2) b	<0.0001

Data are presented as mean (with the range). Different letters in consecutive cells were statistically different when *p* < 0.05. PWB—Polish warmblood; sharp e.—sharp enamel points; overgrown t.—overgrown tooth; wave m.—wave mouth; step m.—step mouth; displaced t.—displaced teeth; RDT—retained deciduous teeth; ETR—excessive transverse ridges.

**Table 10:** The frequency distribution of dental diseases of premolar * and molar ** teeth in the total 7912 examined teeth of 206 horses.

	Loose t. *	Fractures *	Caries *	Calculus *	*p*	Loose t. **	Fractures **	Caries **	Calculus **	*p*
Total	0.04 (2) a	0.02 (1) a	0.07 (2) a	0 (0) a	0.059	0.04 (5) a	0.06 (2) ab	0.14 (2) b	0 (0) a	<0.0001
0–5 years	0 (0)	0 (0)	0 (0)	0 (0)	nc	0 (0)	0 (0)	0 (0)	0 (0)	nc
6–10 years	0 (0) a	0 (0) a	0.05 (2) b	0 (0) a	0.028	0 (0) a	0.03 (1) a	0.04 (2) a	0 (0) a	0. 257
11–15 years	0.02 (1) a	0.02 (1) a	0.14 (2) a	0 (0) a	0.098	0 (0) a	0.09 (2) ab	0.16 (2) b	0 (0) a	0.004
>15 years	0.13 (2) a	0.07 (1) ab	0.04 (2) a	0 (0) b	0.036	0.17 (5) ab	0.11 (2) ab	0.30 (2) a	0 (0) b	0.003
Male	0.03 (1) a	0.02 (1) a	0.07 (2) a	0 (0) a	0.185	0.02 (1) a	0.04 (2) a	0.14 (2) b	0 (0) a	0.0002
Female	0.05 (2) a	0.03 (1) a	0.06 (2) a	0 (0) a	0.378	0.09 (5) ab	0.10 (2) ab	0.14 (2) a	0 (0) b	0.028
PWB	0.05 (1) a	0.05 (1) a	0.06 (2) a	0 (0) a	0.175	0.03 (1) a	0.04 (2) a	0.17 (2) b	0 (0) a	<0.0001
Pony	0.05 (2) a	0 (0) a	0.10 (2) a	0 (0) a	0.294	0.15 (5) a	0.05 (1) a	0.10 (2) a	0 (0) a	0.559
“Other”	0.02 (1) a	0 (0) a	0.05 (2) a	0 (0) a	0.294	0 (0) a	0.12 (2) a	0.08 (2) a	0 (0) a	0.051

Data are presented as mean (with the range). Different letters in consecutive cells were statistically different when *p* < 0.05. PWB—Polish warmblood. Different superscripts in consecutive cells were statistically different. Loose t.—loose teeth; nc—not calculable.

## Data Availability

The data presented in this study are available on request from the corresponding author.

## Supplementary Materials

The following are available online at https://www.mdpi.com/article/10.3390/ani12223120/s1. Figure S1: An equine dental chart used during the detailed dental examination, Table S1: The definitions of selected disorder (malocclusions and dental diseases) of the incisor teeth, Table S2: The definitions of selected disorder (malocclusion and dental diseases) of the canine teeth, Table S3: The definitions of selected disorder (malocclusion and dental disease) of the wolf teeth, Table S4: The definitions of selected disorder (malocclusions and dental diseases) of the cheek (premolar and molar) teeth.
