# Supplementary material for: Malocclusions and Dental Diseases in Privately Owned Horses in the Mazovia Region of Poland
Source: Animals (Basel). 2022 Nov 11;12(22):3120. doi: 10.3390/ani12223120 (PMC9686654; doi:10.3390/ani12223120)
Supplement: Supplementary file 1 [file animals-12-03120-s001.zip › animals-1955639-supplementary.pdf]

Supplementary Material

Malocclusions and Dental Diseases in Privately Owned Horses in the Mazovia Region of Poland

Kamil Górski [1,*], Elżbieta Stefanik [1], Bernard Turek [1], Andrzej Bereznowski [2], Michał Czopowicz [2], Izabela Polkowska [3] and Małgorzata Domino [1,*]

[1] Department of Large Animal Diseases and Clinic, Institute of Veterinary Medicine, Warsaw University of Life Sciences, 02-787 Warsaw, Poland
[2] Department of Veterinary Epidemiology and Economics, Faculty of Veterinary Medicine, Warsaw University of Life Sciences, 02-787 Warsaw, Poland
[3] Department and Clinic of Animal Surgery, Faculty of Veterinary Medicine, University of Life Sciences in Lublin, 20-950 Lublin, Poland
* Correspondence: kamil_gorski@sggw.edu.pl (K.G.); malgorzata_domino@sggw.edu.pl (M.D.); Tel.: +48-22-593-61-91 (M.D.)

EQUINE DENTAL CHART

Date	Name	Age	Sex	Breed
Color	Use	Last exmination		
Owner		History		
Adress			Phone	

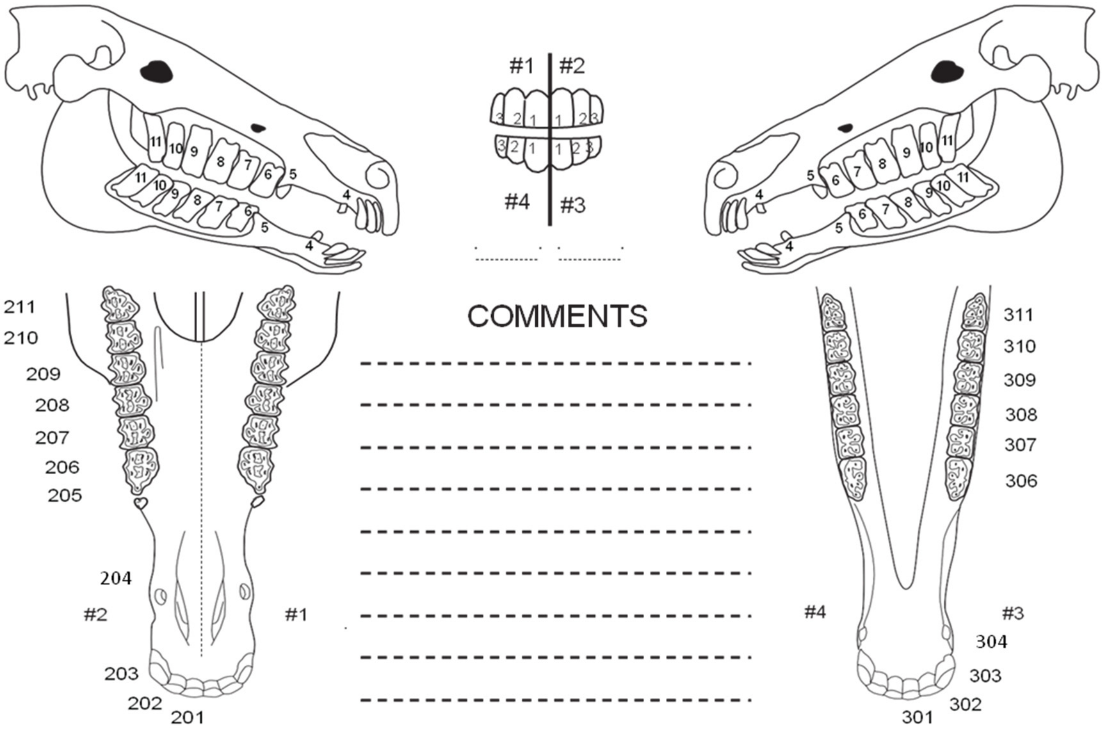

INCISORS	CANINES	WOLF TEETH	PREMOLARS/ MOLARS	
▫ Underbite/Underjet	▫ Normal	▫ Present	▫ Normal	▫ Cap
▫ Overbite/Overjet	▫ Missing	▫ Missing	▫ Sharp points	▫ Cap fragments
▫ Ventral curvature	▫ Shortened	▫ Blind	▫ Wave	▫ Angled
▫ Dorsal curvature	▫ Rounded	▫ Extract	▫ Stepped	▫ Mobile
▫ Slant curvature	▫ Calculus	▫ Other	▫ Shear	▫ Protuberant
▫ Irregular curvature	▫ Fracture		▫ Hooks	▫ Supernumerary
▫ Fracture	▫ Blind		▫ ETR	▫ Missing
▫ Supernumerary	▫ Periodontitis		▫ Erosion / Ulcers	▫ Fracture
▫ Missing	▫ Other			▫ Other
▫ Calculus			▫ Periodontitis -	Stage 1 2 3 4
▫ Periodontitis				
▫ Other				

Sedation: .... Re-examination recommended: ……………..........................

Figure S1. An equine dental chart used during the detailed dental examination.

Table S1. The definitions of selected disorder (malocclusions and dental diseases) of the incisor teeth.

Name	Definition
	Malocclusions
underbite	an occlusal aspects of the mandibular incisors lie rostral to the occlusal aspects of the maxillary incisors, with contact between teeth [45];
overbite	an occlusal aspects of the maxillary incisors lie rostral to the occlusal aspects of the mandibular incisors, with contact between teeth [45]. Protrusion of the maxillary incisor teeth rostrally and horizontally beyond the limits of the mandibular incisor teeth from centric occlusion [42];
curvature	an irregularities of the occlusal surface of the incisors: the occlusal line formed by the maxillary and mandibular incisors should lie in a horizontal plane, parallel to the ground. All other configurations, i.e. ventral, dorsal, diagonal, and irregular curvature are considered as abnormal [43,44];
diastema	a diastema is defined as a detectable abnormal interdental (interproximal) space between 2 adjacent teeth [57] with or without food impaction and may be the cause of painful dental disease in horses [57];
oligodontia	a reduced number of teeth. Can be developmental, as a result of an inappropriate differentiation of dental lamina and tooth germ, or may occur during the animal's lifetime, due to traumatic causes, age, or secondary to other dental disorders [45];
polydontia	a presence of teeth in excess of the normal dental formula, as a result of an inappropriate differentiation of dental germinal tissue during gestational development, with external trauma also acting as an initiating factor, when teeth germs are affected [45];
RDT	a tooth remaining in the oral cavity longer than physiologically appropriate. After this period, the milk tooth (incisor, premolar) remaining in the oral cavity is considered to be persistent [57];
EOTRH	a teeth-damaging and pain-causing, mainly affecting a horse's incisors and less frequently their canines, premolars, and molars. The etiopathogenesis of the illness has not been fully recognized [53];
hooks	a protuberant crown longer than it is wide [50].
	Dental diseases
supernumerary	a presence of an extra incisor tooth in excess of the normal number for horses [46];
loose teeth	a very pronounced, excessive tooth mobility, most often associated with the presence of diastema, advanced periodontitis, and accumulation of food contents [46,50];
fractures	a typically caused by traumatic injuries, affecting some or all of the tooth fragments (clinical crown, reserve crown, root) [57];
caries	a destruction of infundibular calcified dental tissues, due to demineralization and bacterial infection (erosions and dark discolouration) of its cementum with or without extension of caries to the enamel and dentine [57];
calculus	an accumulation of tartar; occurs as a result of the mineralization of the bacterial plaque flora on the teeth [50].

RDT - retained deciduous teeth; EOTRH - Equine Odontoclastic Tooth Resorption and Hypercementosis syndrome.

Table S2. The definitions of selected disorder (malocclusion and dental diseases) of the canine teeth.

Name	Definition
	Malocclusion
non-erupted canines	a tooth which has not protruded above the gingival surface and the horse is 6 years old [57].
	Dental diseases
fractures	a typically caused by traumatic injuries, affecting some or all of the tooth fragments (clinical crown, reserve crown, root) [57];
calculus	an accumulation of tartar; occurs as a result of the mineralization of the bacterial plaque flora on the teeth [50].

Table S3. The definitions of selected disorder (malocclusion and dental disease) of the wolf teeth.

Name	Definition
	Malocclusion
blind wolf tooth	a hidden wolf tooth, first premolar referred to as a brachydont tooth and considered vestigial, as it failed to molarize during evolution [46].
	Dental disease
fractures	a typically caused by traumatic injuries, affecting some or all of the tooth fragments (clinical crown, reserve crown, root) [57].

Table S4. The definitions of selected disorder (malocclusions and dental diseases) of the cheeck (premolar and molar) teeth.

Name	Definition
	Malocclusions
sharp enamel points	a presence of protruding enamel points on the buccal edges of maxillary teeth and lingual edges of the mandibular teeth, which develop as a result of anisognathism and the equid's masticatory action [57];
overgrow tooth	a tooth that has not been exposed to normal wear, most commonly secondary to loss or excessive wear of the opposing tooth; involves individual teeth [55,57];
wave mouth	an unequal or corrugated appearance of the occlusal surface in a rostrocaudal direction or more than one tooth with excess crown in a single arcade [46,50];
step mouth	a supra-eruption, classically rectangular shape of individual teeth, usually opposite to a missing teeth [14];
displaced teeth	a variation of the physiological position inside the teeth arch. Tooth displaced in a buccal (premolars and molars) or distal (incisors) direction [22,46];
diastema	a diastema is defined as a detectable abnormal interdental (interproximal) space between 2 adjacent teeth [57] with or without food impaction and may be the cause of painful dental disease in horses [57];
oligodontia	a reduced number of teeth. Can be developmental, as a result of an inappropriate differentiation of dental lamina and tooth germ, or may occur during the animal's lifetime, due to traumatic causes, age, or secondary to other dental disorders [45];
polydontia	a presence of teeth in excess of the normal dental formula, as a result of an inappropriate differentiation of dental germinal tissue during gestational development, with external trauma also acting as an initiating factor, when teeth germs are affected [45];
RDT	a tooth remaining in the oral cavity longer than physiologically appropriate. After this period, the milk tooth (incisor, premolar) remaining in the oral cavity is considered to be persistent [55,57];
ETR	a transverse edges on the rubbing surface of the tooth which are at least 3 mm in height [50]. ETR may lead to more severe dental diseases such as diastema and periodontal disease because of the ridges forcing feed interproximally in

	the opposing arcade during mastication [55];
hooks	a protuberant crown longer than it is wide [50]; it is most commonly found on teeth 06 or 11 and is often associated with a mismatch between the length of the occlusal surfaces of the maxilla and mandible (undershot or overshot bite) [46].
	Dental diseases
loose teeth	a very pronounced, excessive tooth mobility, most often associated with the presence of diastema, advanced periodontitis, and accumulation of food contents [46,50];
fractures	a typically caused by traumatic injuries, affecting some or all of the tooth fragments (clinical crown, reserve crown, root) [57];
caries	a destruction of infundibular calcified dental tissues, due to demineralization and bacterial infection (erosions and dark discolouration) of its cementum with or without extension of caries to the enamel and dentine [57];
calculus	an accumulation of tartar; occurs as a result of the mineralization of the bacterial plaque flora on the teeth [50].

RDT - retained deciduous teeth; ETR - excessive transverse ridges.
